# The Mechanical Treatment of Neuralgia

**Published:** 1883-02

**Authors:** 


The Mechanical Treatment of Neuralgia.—The author (Dr. E. Rasori) uses the tuning-fork in the treatment of neuralgic pains, applying its vibrating over the course of the painful nerves. He reports the experiments of Boudet, who, by means of the tuning-fork, could check a neuralgia for some time. Boudet used the instrument in accordance with the ideas of Granvill, who thought the neuralgia consisted in a peculiar vibration in the nerve trunk, to induce different vibrations in the painful nerves. He mentions many other experiments from Bal, of Paris, and Renzer and Growers, of London, where the application of the instrument was of benefit.

The instrument was applied for from twenty to forty minutes, when the patient was relieved without further treatment. During the neuralgic attacks, one of the women had suffered from vomiting, but after the relief from the application, she was troubled no more in this way. —*Bolletino della Societa Hancisiana, Roma.*

